# Overexpression of the Oral Mucosa-Specific microRNA-31 Promotes Skin Wound Closure

**DOI:** 10.3390/ijms20153679

**Published:** 2019-07-27

**Authors:** Lin Chen, Alyne Simões, Zujian Chen, Yan Zhao, Xinming Wu, Yang Dai, Luisa A. DiPietro, Xiaofeng Zhou

**Affiliations:** 1 Center for Wound Healing & Tissue Regeneration, College of Dentistry, University of Illinois at Chicago, Chicago, IL 60612, USA; 2 Department of Periodontics, College of Dentistry, University of Illinois at Chicago, Chicago, IL 60612, USA; 3 Oral Biology Laboratory, Department of Biomaterials and Oral Biology, School of Dentistry, University of São Paulo, 05508-000 São Paulo, SP, Brazil; 4 Department of Bioengineering, College of Engineering, University of Illinois at Chicago, Chicago, IL 60607, USA; 5 Graduate College, University of Illinois at Chicago, Chicago, IL 60607, USA; 6 UIC Cancer Center, University of Illinois at Chicago, Chicago, IL 60612, USA

**Keywords:** wound-healing, microRNA, miR-31, oral mucosal wound, skin wound

## Abstract

Wounds within the oral mucosa are known to heal more rapidly than skin wounds. Recent studies suggest that differences in the microRNAome profiles may underlie the exceptional healing that occurs in oral mucosa. Here, we test whether skin wound-healing can be accelerating by increasing the levels of oral mucosa-specific microRNAs. A panel of 57 differentially expressed high expresser microRNAs were identified based on our previously published miR-seq dataset of paired skin and oral mucosal wound-healing [Sci. Rep. (2019) 9:7160]. These microRNAs were further grouped into 5 clusters based on their expression patterns, and their differential expression was confirmed by TaqMan-based quantification of LCM-captured epithelial cells from the wound edges. Of these 5 clusters, Cluster IV (consisting of 8 microRNAs, including miR-31) is most intriguing due to its tissue-specific expression pattern and temporal changes during wound-healing. The in vitro functional assays show that ectopic transfection of miR-31 consistently enhanced keratinocyte proliferation and migration. In vivo, miR-31 mimic treatment led to a statistically significant acceleration of wound closure. Our results demonstrate that wound-healing can be enhanced in skin through the overexpression of microRNAs that are highly expressed in the privileged healing response of the oral mucosa.

## 1. Introduction

Wound-healing in adult tissues is a complex event that generally ends with a scar. The elimination of scar formation remains an elusive goal in the wound repair field. One tissue that exhibits rapid wound-healing with reduced scar formation is the oral mucosa. Studies in at least 3 different models (human, pig and mouse) support that enhanced wound closure is a shared feature of oral mucosal tissue across species [1,2,3,4]. It has been suggested that differences in the physical environment and inflammatory responses at particular anatomical sites may lead to site-specific variations in the wound-healing process. Yet, skin transplanted into oral cavity maintains its morphology [5], and healing properties [6], suggesting that site-specific injury responses are likely to involve intrinsic elements rather than arising simply from environmental factors. Our prior studies clearly demonstrate functional differences in the injury response between epithelial cells from skin and oral mucosa [1,2,7,8]. For example, compared to the epithelial cells from skin, oral mucosal epithelial cells exhibit enhanced proliferation and migration in vitro, which parallels the rapid wound-healing observed in oral mucosa in vivo. These results imply that the site-specific wound-healing phenotype involves intrinsic modifications in the response of epithelial cells to a wound. Such findings suggest that skin wound-healing might be improved by genetic modification with elements specific to regenerative oral mucosal repair. 

To date, the available evidence clearly reveals the complexity of the highly orchestrated wound-healing process at the genomic and epigenomic levels. Our previous studies provided the dynamic time course-based profiles of both the transcriptome and microRNAome on a paired oral mucosa and skin wound-healing model, and showed that site-specific injury responses exist at each site [9,10]. Studies focused on individual microRNA genes have demonstrated several important roles for microRNAs in wound-healing. For instance, miR-21, an oncomir identified in many cancers of epithelial origin, promotes keratinocyte migration and re-epithelialization in wound-healing [10,11,12,13]. On the other hand, miR-10b negatively regulate the wound closure by suppressing the proliferation and migration of epithelial cells [10]. The expression of miR-99 family members (miR-99a, miR-99b, miR-100) is co-regulated during skin wound-healing, and these microRNAs in turn contribute to injury response by regulating the IGF1R-AKT-mTOR signaling [14,15]. Together, these findings demonstrate the critical functional role of microRNAs in the wound-healing process. In this study, we identify and validate the microRNA patterns associated with the rapid wound closure in oral mucosa, and we also test the feasibility of promoting skin wound closure by introducing oral mucosal wound-associated microRNA changes in skin wounds. Our results demonstrated that overexpression of miR-31, a microRNA that is highly expressed in mucosa, accelerates the closure of skin wounds.

## 2. Results

### 2.1. Specific Patterns of Highly Expressed MicroRNAs in Skin and Oral Mucosal Wound-Healing

**Figure 1:**
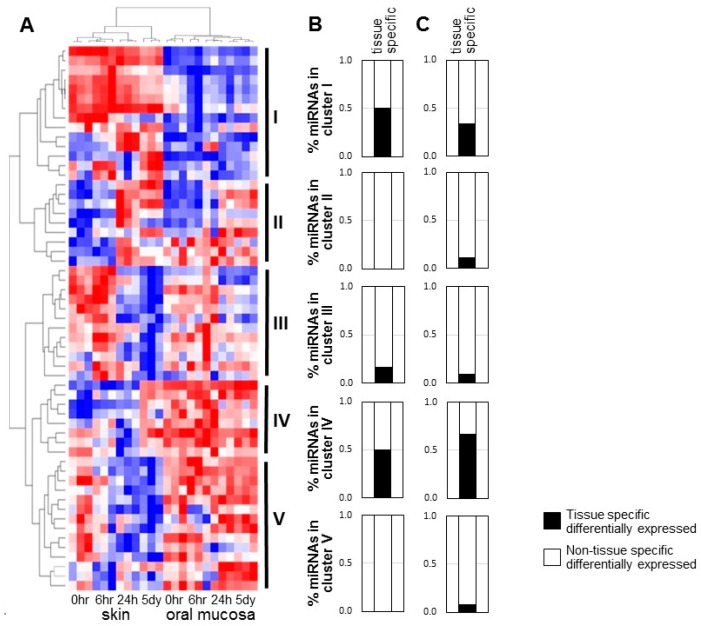
Differentially expressed high expresser microRNAs during wound-healing of skin and oral mucosal epithelium. An existing miR-Seq dataset of paired skin and oral mucosal wound-healing over time (0 h, 6 h, 24 h, 5 day) was reanalyzed and a cutoff value of 5458 (average number of mapped sequence reads of the whole dataset) was applied. A panel of 57 differentially expressed (*p* < 0.01) highly expressed microRNAs were identified, which were grouped into 5 clusters based on hierarchical clustering (**A**). The tissue specificity of these clusters was further assessed by comparing the members of each clusters with a previously reported panel of tissue-specific differentially expressed microRNAs [10]. The percentage of microRNAs that were identified as tissue specific in each cluster is presented (**B**). An independent microRNA profiling analysis of unwounded skin and oral mucosal epithelium (base line, *n* = 3, pooled samples), performed using a mouse microRNA microarray, allowed the identification of 85 tissue-specific microRNAs (Supplementary Figure S4 and Supplementary Table S2). The percentage of microRNAs in each of the 5 clusters that were identified as tissue specific based on this new list is shown (**C**).

We previously explored the differences in the miRNome during the time course of skin and oral mucosal wound-healing by miR-Seq analysis [10], and found that highly expressed microRNAs account for the majority of miRNome in both skin and oral mucosa during wound-healing. Specifically, highly expressed microRNAs [with number of mapped sequence reads greater than the average (5458) of the miR-Seq dataset, 12.3% and 13.1% of the unique microRNA species in skin and oral mucosa, respectively] account for 94.76% and 92.66% of the miRNomes, respectively (Supplementary Figure S1). The low expressing microRNA species (89.69% and 86.86% of the unique microRNA species in skin and oral mucosa, respectively) account for a very small portion of the miRNomes, and tend to exhibit high probability of sequencing errors (Supplementary Figure S2). To focus on defining the biological relevance of the highly expressed microRNAs in the differential injury response in skin and oral wound-healing, we reanalyzed our existing miR-Seq dataset and identified 57 differentially expressed (*p* < 0.01) highly expressed microRNAs [number of mapped sequence reads greater than the average (5458) of the dataset] (Figure 1A and Supplementary Table S1). These 57 microRNAs account for 73.4% and 64.5% of the miRNomes in skin and oral mucosa epithelium (*p* = 0.001), with these fractions remaining relatively constant during the time course of the wound-healing (*p* = 0.544 and *p* = 0.975, respectively (Supplementary Figure S3). Unsupervised classification (hierarchical cluster analysis) of the 57 differentially expressed highly expressed microRNAs revealed 5 clusters of microRNAs. The microRNAs grouped in Cluster I exhibit higher overall expression levels in skin than in oral mucosal wounds, whereas Cluster IV and V microRNAs exhibit higher overall levels in oral mucosal wounds as compared to skin wounds. The expression patterns of Cluster II and III microRNAs are similar between skin and oral wounds. Cluster II consists of microRNAs that increased during both skin and oral wound-healing, whereas Cluster III consists of microRNAs that were down-regulated during both skin and oral wound-healing.

To assess the tissue specificity, the microRNAs from these 5 hierarchical clusters were matched with our previously reported panel of 53 tissue-specific differentially expressed microRNAs [10]. As showed in Figure 1B, Cluster I and IV contain significantly enriched amounts of tissue-specific microRNAs (approximately 50.0%, chi-square test *p* = 0.005 and *p* = 0.048, respectively). In contrast, Cluster II and V do not contain any tissue-specific microRNAs, while Cluster III contains 16.7% tissue-specific microRNAs. To confirm the tissue specificity, an independent microRNA profiling analysis was performed on baseline unwounded skin and oral mucosal epithelium using a mouse microRNA microarray; this analysis identified 85 tissue-specific differentially expressed microRNAs (Supplementary Figure S4 and Supplementary Table S2). When compare this newly acquired set of 85 tissue-specific microRNAs to the 5-cluster of the differentially expressed highly expressed microRNAs, Cluster IV contained the most tissue-specific microRNAs (66.7%) as compared to other clusters (I: 33.3%, II: 11.1%, III: 9.1%, V: 7.7%) (Figure 1C, and Supplementary Table S3). The enrichment in Cluster IV was statistically significant (chi-square test *p* = 0.003). Thus, of the 5 clusters, Cluster IV contains a group of tissue-specific and wound-healing associated microRNAs and was therefore subjected to additional validation studies. 

### 2.2. Validation of microRNA Differential Expression in LCM-Captured Epithelial Cells from Skin and Oral Mucosal Wounds

**Figure 2:**
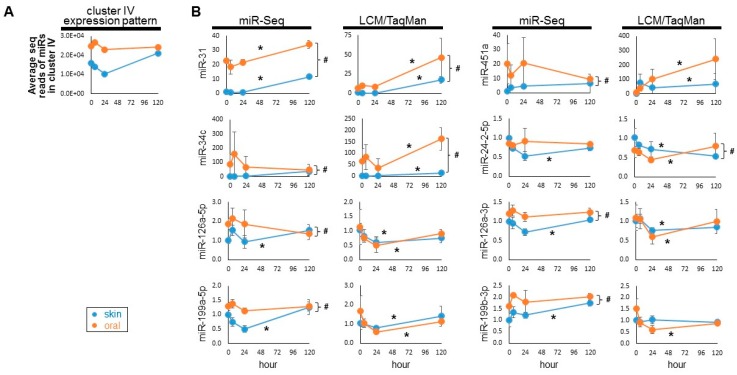
Validation of the differentially expressed microRNAs in Cluster IV. (**A**) The general expression pattern of Cluster IV over the time course of skin and oral mucosal wound is shown as the average of the mapped sequence reads of the microRNAs in the cluster. (**B**) LCM isolated epithelial cells from the wound edges were procured from the paired skin and oral mucosa murine wound-healing model, at 0 h, 6 h, 24 h and 5 days post-wounding (n ≥ 4). The levels of individual Cluster IV microRNAs (mmu-miR-31-5p, mmu-miR-451a, mmu-miR-34c-5p, mmu-miR-24-2-5p, mmu-miR-126a-5p, mmu-miR-126a-3p, mmu-miR-199a-5p, mmu-miR-199b-3p) were assessed by TaqMan assay-based real time PCR quantification (in duplicates). Expression patterns were compared with the relative levels of these microRNAs measured previously by miR-seq [10]. * indicates one-way ANOVA test *p*-value < 0.05 (within series comparison); ^#^ indicates two-way ANOVA test *p*-value < 0.05 (between series comparison). Statistical analyses are presented in Supplementary Table S4.

Cluster IV consists of 8 microRNAs (mmu-miR-31-5p, mmu-miR-451a, mmu-miR-34c-5p, mmu-miR-24-2-5p, mmu-miR-126a-5p, mmu-miR-126a-3p, mmu-miR-199a-5p, mmu-miR-199b-3p). Examination of the general expression pattern of this 8-microRNA cluster (represented as the average of the mapped sequence reads of the microRNAs in the cluster) showed that these microRNAs in this cluster generally exhibit relatively high expression levels in oral mucosa as compared to skin (Figure 2A). While their levels remain relatively constant during oral mucosal wound-healing, an early decrease followed by a late increase was observed in skin wound-healing. To confirm the differential expression of these Cluster IV microRNAs, LCM was performed to selectively capture epithelial cells from the wound edges of both skin and oral mucosal wounds at 6 h, 24 h and 5 days post-wounding, and compared with unwounded baseline LCM-captured cells (0 h). TaqMan assay-based validation was performed on RNA from the LCM-captured cells to quantify the Cluster IV microRNAs, and the results were compared to the miR-Seq-based quantification of each microRNAs (Figure 2B, and Supplementary Table S4). While similar expression patterns were observed between miR-seq quantification results and TaqMan/LCM validation data for all 8 Cluster IV microRNAs, miR-31 exhibited the most consistency between miR-Seq and LCM/TaqMan analysis—differential expression was detected for both the individual time course (one-way ANOVA *p*-value < 0.05) and between time course series (two-way ANOVA *p*-value < 0.05).

**Figure 3:**
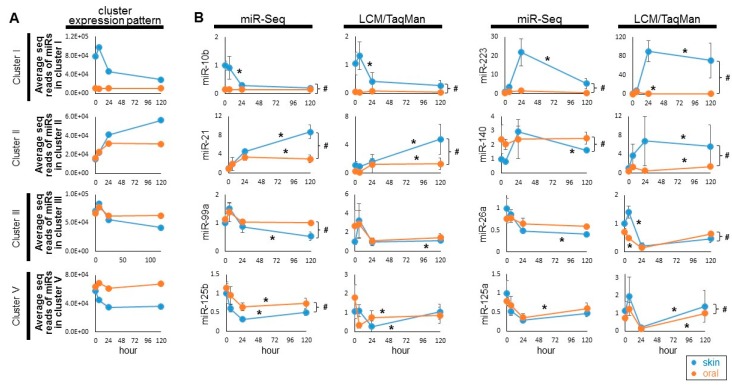
Validation of the selected differentially expressed microRNAs in Cluster I, II, III, and V. (**A**) The general expression pattern of Cluster I, II, III, and V during the paired skin and oral mucosal wound-healing time course was represented as the average of the mapped sequence reads of the microRNAs in the cluster. (**B**) LCM isolated epithelial cells from the wound edges were procured from the paired skin and oral mucosa murine wound-healing model, at 0 h, 6 h, 24 h and 5 days post-wounding (*n* ≥ 4). The levels of selected microRNAs from Cluster I (mmu-miR-10b, mmu-miR-223), Cluster II (mmu-miR-21, mmu-miR-140), Cluster III (mmu-miR-99a, mmu-miR-26a), and Cluster V (mmu-miR-125b, mmu-miR-125a) were assessed by TaqMan assay-based real time PCR quantification (in duplicates). The expression patterns were compared with the relative levels of these microRNAs measured previously by miR-seq [10]. * indicates one-way ANOVA test *p* < 0.05 (within series comparison); ^#^ indicates two-way ANOVA test *p* < 0.05 (between series comparison). Statistical analyses are presented in Supplementary Table S4.

TaqMan assay-based validation with LCM-captured cells was also performed on selected microRNAs from Cluster I (mmu-miR-10a-5p, mmu-miR-223-3p), Cluster II (mmu-miR-21-5p, mmu-miR-140-3p), Cluster III (mmu-miR-99a-5p, mmu-miR-26a-5p) and Cluster V (mmu-miR-125b-5p, mmu-miR-125a-5p) (Figure 3, and Supplementary Table S4). The results were compared to the overall expression pattern of specific clusters (represented as the average of the mapped sequence reads of the microRNAs in the cluster) (Figure 3A), as well as the miR-Seq-based quantification of each individual microRNAs (Figure 3B).

### 2.3. Evaluation of the Functional Contribution of Highly Expressed microRNAs in Wound-Healing

**Figure 4:**
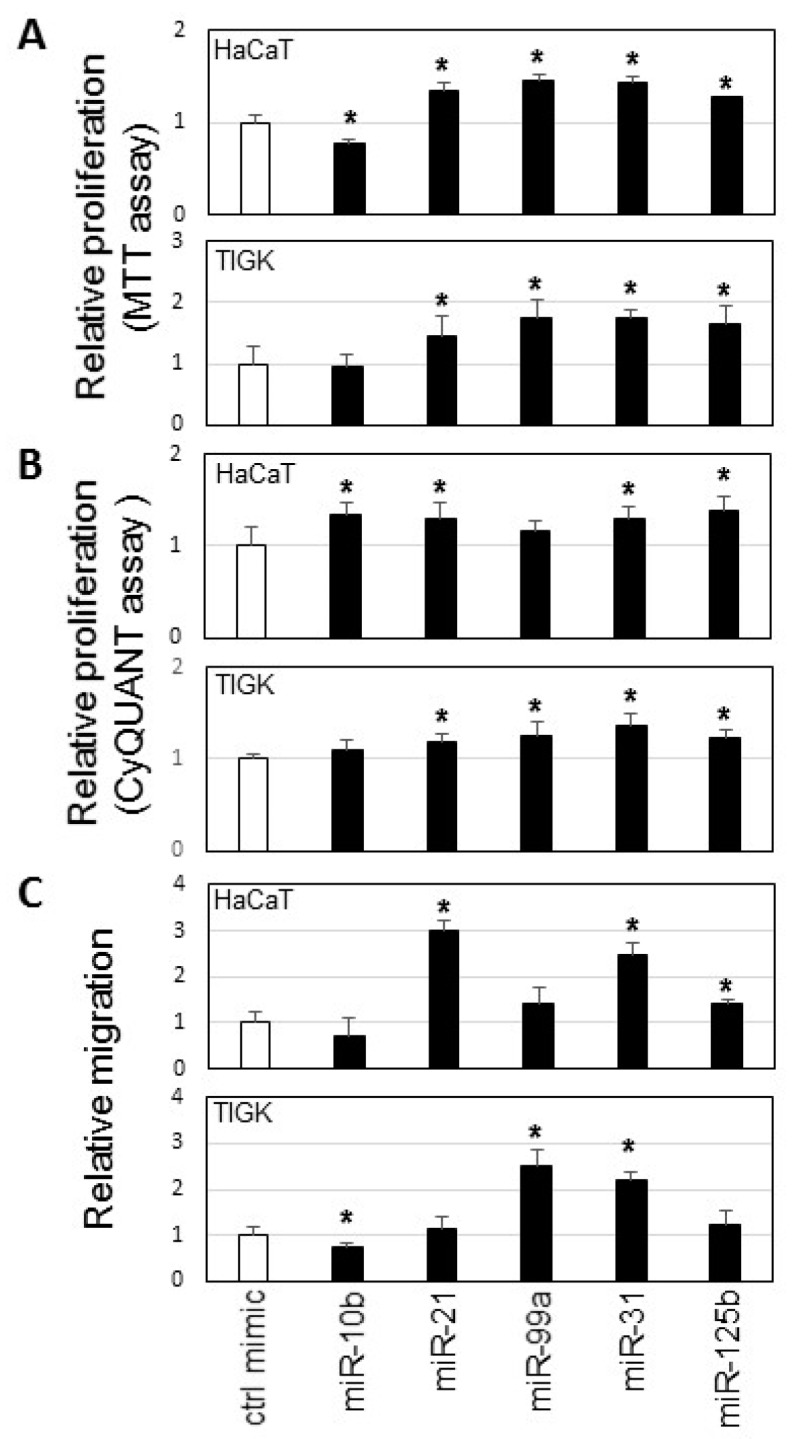
MicroRNA effects on proliferation and cell migration. HaCaT and TIGK cells were transfected with miR-10b mimic, miR-21 mimic, miR-99a mimic, miR-31 mimic, miR-125b mimic, or negative control mimic. MTT assays (**A**), CyQUANT assays (**B**), and cell migration assays (**C**) were performed as described in the Material and Methods section. Data represents at least 3 independent triplicated experiments with similar results (* *p* < 0.05).

We tested the functional effects of several differentially expressed microRNAs from each cluster (cluster I: miR-10b, Cluster II: miR-21, Cluster III: miR-99a, Cluster IV: miR-31, and Cluster V: miR-125b) on wound-healing using in vitro proliferation assays and in vitro migration assay on skin (HaCaT) and oral epithelial (TIGK) cell lines. As showed in Figure 4A,B, ectopic transfection of miR-21, miR-31 and miR-125b consistently enhanced proliferation in both HaCaT and TIGK as measured by 3-[4,5-dimethylthiazole-2-yl]-2,5-diphenyltetrazolium bromide (MTT) assay (based on cell metabolic activity) and CyQUANT assay (based on DNA content). Regarding the in vitro migration assay, only the ectopic transfection of miR-31 consistently enhanced the cell migration of both HaCaT and TIGK (Figure 4C). 

**Figure 5:**
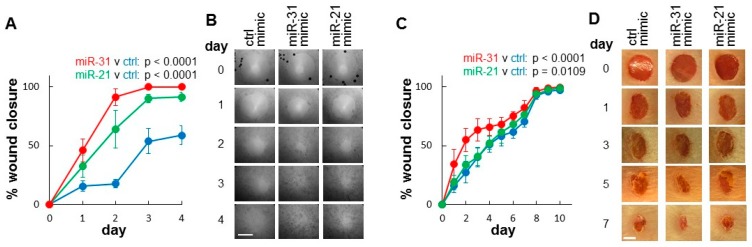
Effect of miR-31 and miR-21 on wound closure. (**A**) HaCaT cells were transfected with miR-31 mimic (red), miR-21 mimic (green), or negative control mimic (blue). In vitro wound closure assays (*n* = 4) were performed as described in the Material and Methods section. *p*-values from two-way ANOVA test (for between time course series comparison) are presented. (**B**) Representative images of in vitro wound closure assays taken at the time points indicated. Scale bar = 1 mm. Data represents at least 3 independent experiments with similar results. (**C**) Wound closure (*n =* 6) was assessed in vivo as described in the Material and Methods section in mouse skin wounds treated with miR-31 mimic (red), miR-21 mimic (green), or negative control mimic (blue) at the time of injury. *p*-values from two-way ANOVA test (for between time course series comparison) are presented in the figure. (**D**) Representative photomacrographs of microRNA mimic-treated wounds taken at the time points indicated. Scale bar = 2 mm.

We then focused on evaluating the effects of miR-31 on wound closure using both in vitro and in vivo wound-healing assays. As showed in Figure 5A,B, ectopic transfection of miR-31 mimic into the skin epithelial cells (HaCaT) accelerated wound closure in an in vitro wound-healing assay when compared to negative control mimic-treated cells. Cells treated with positive control mimic (miR-21, a known enhancer of wound-healing [10,11,12,13]) also exhibited accelerated wound closure. Statistical analyses were presented in Supplementary Table S5. 

We further evaluated the effects of miR-31 in a mouse skin wound-healing model, where an animal origin-free lipid nanoparticle in vivo delivery system was used for introducing the miR-31 mimic to the wounds. As shown in Figure 5C,D, a single dose of miR-31 mimic treatment led to a statistically significant increase in wound closure as compared to wounds treated with negative control mimic. Similarly, and to a lesser extent, a statistically significant acceleration of closure was seen in wounds treated with the positive control (miR-21, a known enhancer of wound-healing [10,11,12,13]). Statistical analyses were presented in Supplementary Table S5. These in vivo results are consistent with the observations from the in vitro wound-healing assays described above. 

## 3. Discussion

Wound-healing is a complex process that is mediated by a highly diverse group of factors, including genomic and epigenomic regulators. Skin and mucosal wound-healing proceed through the same stages (hemostasis, inflammation, proliferation, and remodeling), yet wounds of the oral mucosa heal more quickly and with less scar formation. We recently reported the first dynamic microRNAome profiles of paired skin and oral mucosal wound-healing [10]. Together with our earlier studies of the transcriptome profiles of paired skin and oral mucosal wounds [9], our results suggest that wounds in skin and mucosa exhibit intrinsic differences in their genetic and epigenetic responses to injury.

By focusing on highly expressed microRNAs, our current study defined a panel of differentially expressed microRNAs that are associated with skin and oral mucosal wound-healing. Based on their expression pattern, a cluster of 8 microRNAs was further identified which exhibit tissue specificity. This specific expression pattern was validated by both independent microRNA profiling on additional wound samples and TaqMan-based quantification in LCM-procured epithelial cells from the wound edges. Among these 8 microRNAs (miR-31-5p, miR-451a, miR-34c-5p, miR-24-2-5p, miR-126a-5p, miR-126a-3p, miR-199a-5p, miR-199b-3p), miR-31 and miR-34c have previously been implicated in skin wound-healing [16,17,18]. miR-451a has been implicated in fracture healing in normal and diabetic rats [19,20] and is expressed highly in healing fractures in contrast to non-healing fractures implicating its role in enhanced healing albeit in a different model. miR-24-2-5p is the “passenger strand” or “star strand” of the mature microRNA specie produced from one of the two miR-24 genes in mouse (miR-24-1 and miR-24-2). While the active strand of miR-24 (miR-24-3p) has been showed to regulate epidermal differentiation [21,22], very little is known about miR-24-2-5p. It is worth noting that miR-24-3p is a member of Cluster III, which does not exhibit tissue specificity between skin and oral mucosa. Both strands of the mature miR-126a (miR-126a-5p and miR-126a-3p) and miR-199a (miR-199a-5p and miR-199b-3p; the mature miR-199b-3p sequence is identical to miR-199a-3p in mouse) were grouped into the Cluster IV. While miR-126a has been implicated in diabetic wound-healing [23,24,25], miR-199a has been showed to negatively regulate skin wound angiogenesis [26]. It is possible that miR-199a play a different role in the wound microenvironment, and therefore may have a duel effect in oral mucosal wound-healing. 

Among the microRNAs we tested, the effect of miR-31 on wound closure was demonstrated consistently in both in vitro assays and in vivo wound-healing models. MiR-31 is a well-known tissue-specific regulator in cancer [27] (e.g., tumor suppressor in adenocarcinoma and oncogene in squamous cell carcinoma, including oral squamous cell carcinoma [28,29,30]). While its role has not been previously reported in mucosal wound-healing, several recent studies suggested that miR-31 enhances keratinocyte proliferation and migration, and promotes re-epithelialization during skin wound-healing [16,17]. Studies on other biological systems also suggest miR-31 is a positive regulator for several pro-proliferation and pro-migration pathways, including microtubule associated protein kinase (MAPK) signaling pathway [31,32], Wnt pathway [33,34], HIF-1 hypoxia signaling pathway [35,36,37]. These are consistent with the pathways predicted by bioinformatics methods (Supplementary Table S6). By using a novel animal origin-free lipid nanoparticle-based in vivo delivery system, we were able to achieve miR-31 up-regulation in mouse skin wounds in vivo. While both all wounds closed by day 10 in our murine skin wound model, a statistically significant acceleration of wound closure was observed in the miR-31 treated wounds. The miR-31 treatment wounds exhibit a 50%-closure time of less than 2 days, compared to approximately 4-day 50%-closure time in control group. The time points most affected by the miR-31 overexpression were those from early to midpoint of healing, which overlaps the proliferative phase of skin wound-healing in this model. Additionally, miR-31 may also stimulate wound contraction and thus enhance the wound closure. As such, miR-31 may serve as a novel therapeutic target for promoting wound closure and for the treatment of poorly healing and chronic wounds. As a positive control, we also overexpressed miR-21, a well-established positive wound-healing regulator [12,13,38,39], in the mouse skin wound model, and a statistically significant acceleration in wound closure was observed in the miR-21 overexpression group. 

The effectiveness of microRNA-based application in wound-healing treatment is currently under intense investigation. The microRNA-based therapeutic approaches are thought to have specific advantages over traditional methods. Notably, microRNA treatment can modify several pathways at one time, a feature that is attractive due to the complexity of wound pathology. With proper optimization, this approach may eventually develop into an advanced and relatively inexpensive therapeutic strategy. The novelty of the current study is in our attempt to harness regenerative healing capability from mucosal wounds and to “geno-copy” it into skin wounds. Future studies with impaired wound-healing models (chronic, diabetic, or infected wounds) will be essential to fully explore the translational value of our findings.

## 4. Materials and Methods

### 4.1. Animals and Wound Models

Female Balb/c mice (8–10-week-old, Jackson Laboratory, Bar Harbor, ME, USA) were housed in a temperature-controlled environment (22–24 °C) on a 12 h/12 h light-dark cycle, and were provided with food and water ad libitum. For wound-healing studies in skin and oral mucosa, the previously established mouse excisional skin and oral mucosal wound-healing models were adapted with minor modifications [1,7,10]. In brief, mice were anesthetized, shaved, and cleaned thoroughly with 70% isopropyl alcohol. Two 1 cm long full-thickness incisional wounds were created using a pair of scissors on the dorsal skin of mice (one on each side of the midline), and three 0.5 cm incisional wounds were made on the anterior of the hard palate using a scalpel blade. The animals were sacrificed at 6 h, 24 h and 5 days post-wounding, and the specimens were harvested, placed in RNAlater (Sigma-Aldrich, St. Louis, MO, USA) and stored at −20 °C until use.

### 4.2. In Vivo Treatment with microRNA Mimics 

Mouse skin wounds were treated with microRNA mimics as described previously [10]. In brief, two full-thickness wounds were created with a 5 mm skin biopsy punch (Acu Punch, Acuderm, Ft. Lauderdale, FL, USA) on the dorsal skin of mice. 100 µL of the Invivofectamine 3.0 (Invitrogen, Carlsbad, CA, USA) complex containing 1 nmol of miRIDIAN miR-21 mimic, miR-31 mimic or non-targeting mimic (GE Healthcare Dharmacon, Lafayette, CO, USA) was injected into the surrounding dermis of the wounds at four sites at the time of the injury (day 0). Six animals were used for each treatment groups. The microRNA mimic mediated up-regulation of miR-31 and miR-21 was confirmed by TaqMan assays performed on the wound tissue samples harvested 1 day post-wounding (Supplementary Figure S5). Wounds were photographed daily with a PowerShot SX130 digital camera (Canon, Tokyo, Japan) from a fixed distance with a ruler in field of view. Images were analyzed using AxioVision software (ZEISS, Oberkochen, Germany). The measurements of the two wounds on each animal were averaged. The percent of wound closure was calculated as wound area/original wound area X 100%.

### 4.3. microRNA Profiling

Total RNA (including small RNA) was isolation from matched mouse skin and oral mucosal samples using a miRNeasy Mini Kit (QIAGEN, Hilden, Germany). Pooled skin and oral mucosal samples from three animals were subjected to microRNA profiling using the MRA-1002 miRmouse v21 microarrays (LC Sciences, LLC, Houston, TX, USA) as described previously [14], and the microarray data has been submitted to Gene Expression Omnibus (GEO accession number: GSE125423).

### 4.4. Bioinformatics Analysis

The previously published miR-seq dataset on paired skin and oral mucosal wound-healing time course (GEO accession number: GSE121996) was processed, mapped, and analyzed using a proprietary pipeline script, ACGT101-miR v4.2g (LC Sciences, Houston, TX, USA) as described previously [10]. Student’s *t*-test was used for between group comparisons, one-way ANOVA was used for time course data analysis, and two-way ANOVA was used for between time course series comparison. The Benjamini-Hochberg adjusted *p*-values were computed for multiple hypothesis testing. High expresser microRNA was defined as having the number of seq reads greater than the average of the dataset (5458) at any time point of the wound-healing time course. The first panel of tissue-specific differentially expressed microRNAs was based on this existing miR-seq dataset, where between group comparison made between unwounded skin and oral mucosal epithelium. This tissue-specific microRNA panel was reported in our recent publication [10].

For validation, a second panel of tissue-specific differentially expressed microRNAs was generated based on an independent microRNA profiling analysis on unwounded skin and oral mucosal epithelium using a microRNA microarray. For microarray data analysis, normalization and differential analysis were performed with CyberT [40], using Variance Stabilizing Normalization (VSN) method [41,42]. We chose CyberT due to its capability of handling both microarray and RNA-seq datasets, and its documented superiority in handling small number of replicates [43,44]. Principal Component Analysis (PCA) and hierarchical clustering were carried out with Clustvis [45], and the heatmaps were generated using Morpheus (https://software.broadinstitute.org/morpheus). MicroRNA targeted biological pathways were predicted by DIANA-mirPath (v3.0) [46], and the embedded microT-CDS method [47,48] was used for microRNA target gene prediction (microT threshold = 0.8).

### 4.5. Laser Capture Microdissection (LCM)

Epithelial cells from the wound edges were captured with a LMD7000 Laser Microdissection system (Leica Microsystems, Wetzlar, Germany) as we previously described [49,50].

### 4.6. Real Time PCR

TaqMan MicroRNA Assays (Applied Biosystems, Foster City, CA, USA) were used for the real time PCR-based quantification of microRNA. Relative expression levels of the microRNA were computed with the 2^-delta delta Ct^ method [51], where snRNA U6 was used as internal reference.

### 4.7. Cell Culture and in Vitro Functional Assays

Human keratinocytes HaCaT (ATCC, Manassas, VA, USA) and TIGK (a gift from Dr Richard J. Lamont, University of Louisville) were grown as previously described [15,52]. For functional analysis, miRIDIAN microRNA mimics (miR-21 mimic, miR-31 mimic, or non-targeting miRNA mimic, GE Healthcare Dharmacon, Lafayette, CO) were transfected into cells using DharmaFECT Transfection Reagent 1 as described previously [53,54,55]. Cell proliferation was assessed using both MTT assay and a CyQUANT Direct Cell Proliferation Assay (Invitrogen/Molecular Probes) as described previously [14,56]. In vitro cell migration was measured using the Oris 96-well cell migration assay kit (Platypus Technologies, Fitchburg, WI, USA) on cells pre-treated with mitomycin C (10 μg/mL, Sigma-Aldrich, for 2 h) to prevent cell proliferation as described previously [10,57]. In vitro wound closure assay was performed using the Oris 96-well cell migration assay kit (Platypus Technologies, Fitchburg, WI, USA). In brief, cells were seeded in the trans-wells and cultured to confluence. Circler excisions of 2 mm in diameter were created by the removal of the stoppers, and cells were washed and then incubated in complete medium containing cell growth supplements. Wound closure was monitored for 4 days using a digital camera, and the images were analyzed using ImageJ analysis software [version 1.421, National Institutes of Health (NIH)].

## Supplementary Materials

Supplementary materials can be found at https://www.mdpi.com/1422-0067/20/15/3679/s1.
