# Supplementary material for: Overexpression of the Oral Mucosa-Specific microRNA-31 Promotes Skin Wound Closure
Source: Int J Mol Sci. 2019 Jul 27;20(15):3679. doi: 10.3390/ijms20153679 (PMC6696114; doi:10.3390/ijms20153679)
Supplement: Supplementary file 1 [file ijms-20-03679-s001.pdf]

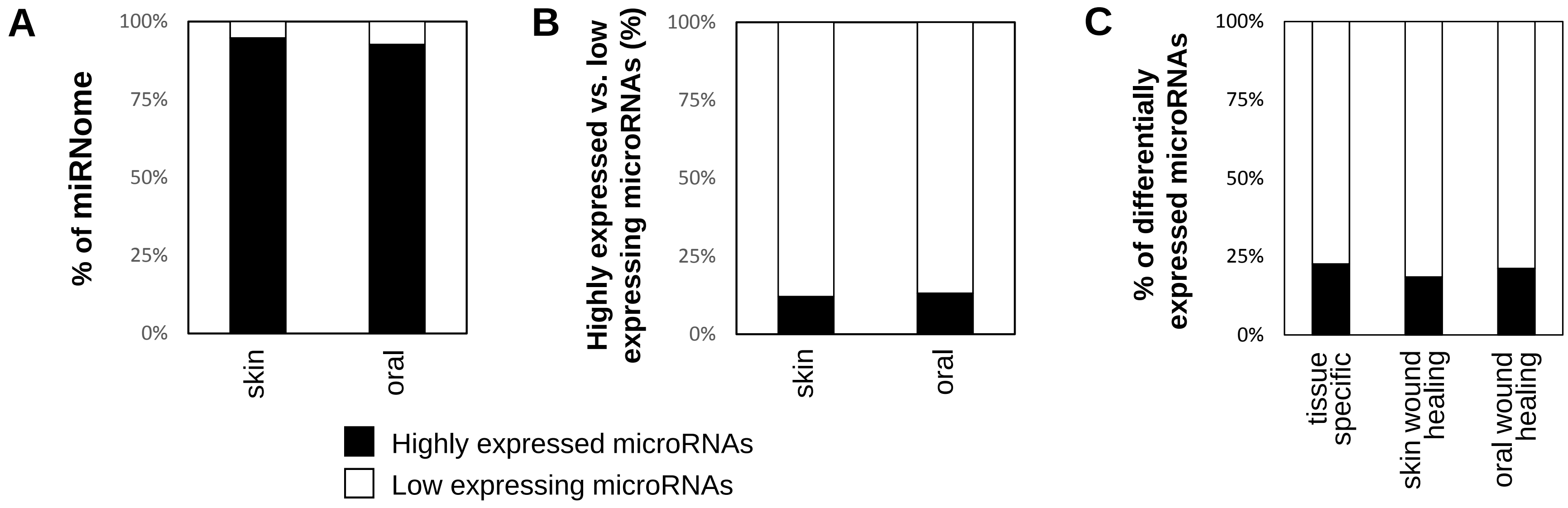

Supplementary Figure S1: The highly expressed microRNAs account for major portion of the miRNome in oral and skin wounds, while the low expressing microRNAs dominant the percentage of unique mapped microRNA species.
A previously reported miR-seq dataset on paired skin and oral mucosal wound healing [Sci. Rep. (2019) 9:7160] was used to examine the relationship of microRNA abundance and the number of unique microRNA species. The highly expressed microRNAs and the low expressing microRNAs are defined using the average number of mapped sequence reads of the overall miR-seq dataset (5458). **A**) The miRNome landscape was presented as histograms of percentage of mapped sequence reads vs. microRNAs grouped by their abundance (highly expressed microRNAs vs. low expressing microRNAs). **B**) The percentage of highly expressed microRNA species and low expressing microRNA species in skin and oral wounds were presented. **C**) Using a p value cut-off of 0.01, 53 are identified as tissue-specific differentially expressed microRNAs; 200 are differentially expressed in skin wound healing; 33 are differentially expressed in oral mucosal wound healing. The percentage of highly expressed microRNAs and low expressing microRNAs in each differentially expressed list is presented.

Supplementary Figure S2: Relationship of sequencing accuracy and number of sequence reads. Phred quality scores on the paired skin and oral mucosal wound healing miR-seq dataset were generated in real time during sequencing by Illumina platforms. A Histogram of the average phred score per base of mappable reads was presented. The phred score is the negative ratio of the error probability. Phred score larger than 30 stands for probability of incorrect base calls less than 1 in 1,000 (above 99.9% accuracy) in one sequencing read.

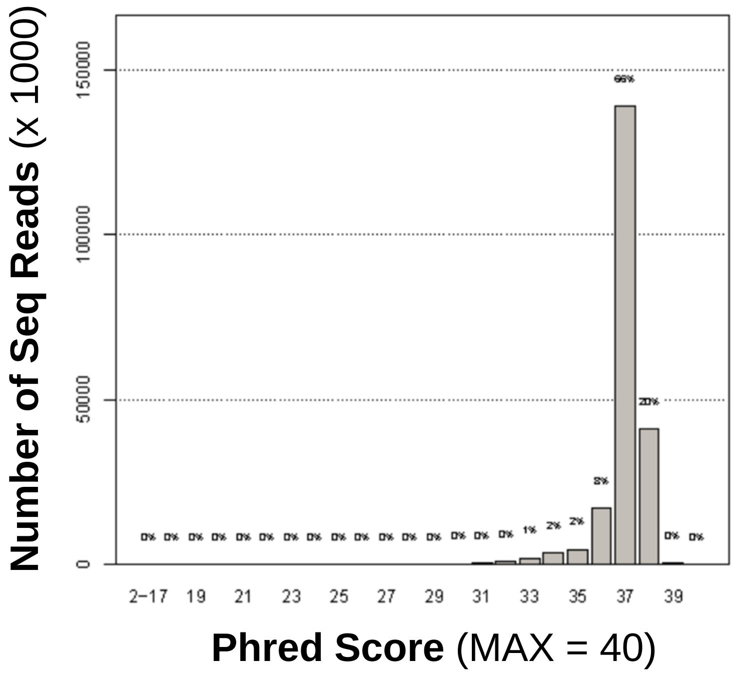

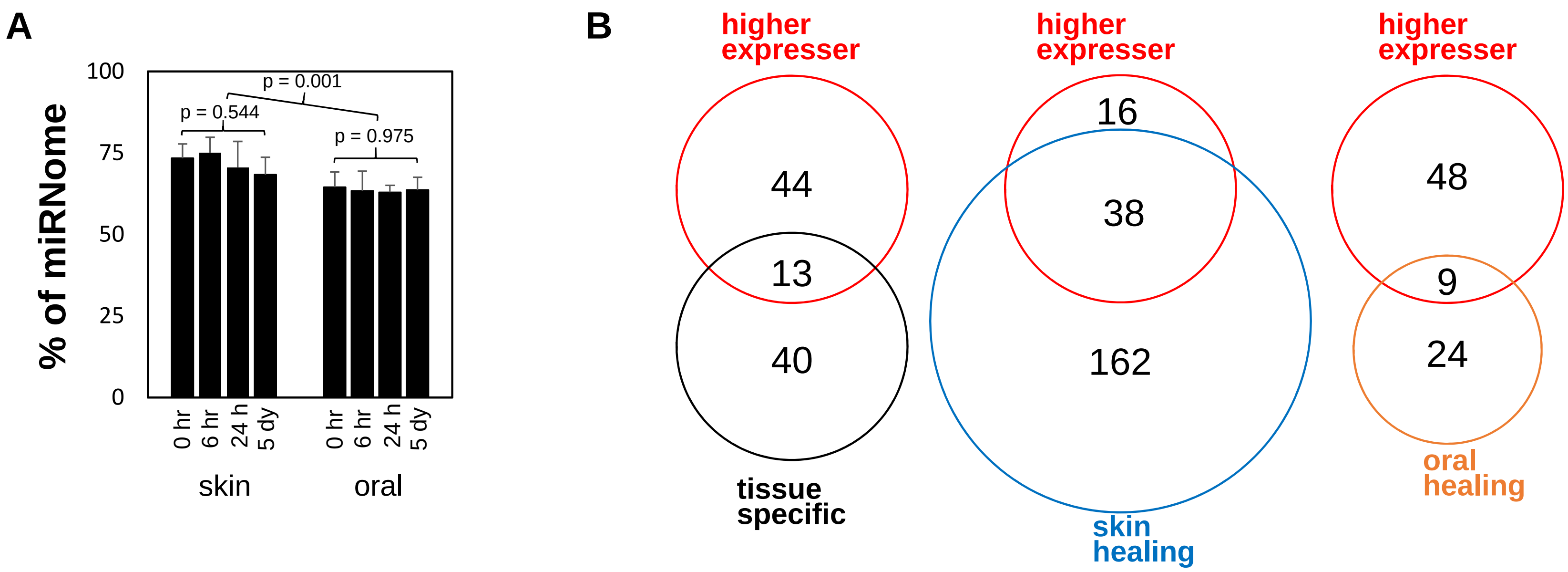

Supplementary Figure S3: Characteristics of the 57 differentially expressed high expresser microRNAs.
(**A**) The 57 differentially expressed high expresser microRNAs account for 73.4% and 64.5% of the miRNomes in skin and oral mucosa epithelium (p = 0.001), and these fractions remaining relatively constant during the time course of the skin and oral mucosal wound healing (p=0.544 and p=0.975, respectively). (**B**) Of the 57 differentially expressed high expresser microRNAs, 13 were previously identified as baseline tissue specific differentially expressed microRNAs, 38 were previously identified as differentially expressed microRNAs associated with skin wound healing, and 9 were previously identified as differentially expressed microRNAs associated with oral mucosal wound healing [Sci. Rep. (2019) 9:7160].

Supplementary Figure S4: Tissue-specific differentially expressed microRNAs in skin and oral mucosal epithelium. The microRNA profiling on matched mouse skin and oral mucosa samples (n=3, pooled) was performed using the MRA-1002 miRmouse v21 microarrays (LC Sciences, Houston, TX). Normalization and differential analysis were performed with CyberT. A panel of 85 tissue-specific differentially expressed microRNAs (q-value<0.01). List of the tissue-specific microRNAs was presented in Supplementary Table S2

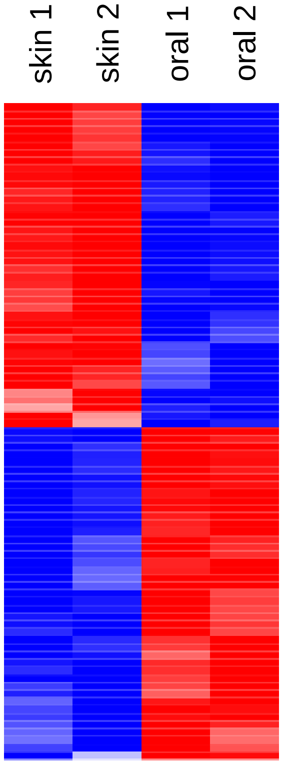

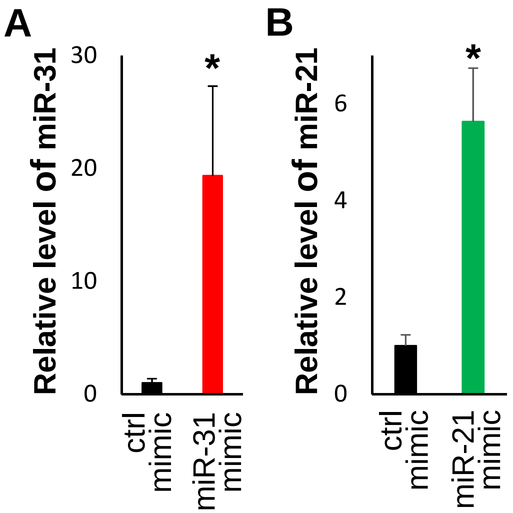

Supplementary Figure S5: Confirmation of the microRNA mimic mediated up-regulation of miR-31 and miR-21 in mouse skin wounds. The miR-31 mimic, miR-21 mimic or negative control mimic were delivered to the mouse skin wounds using an animal origin-free lipid nanoparticle (Invivofectamine 3.0 Reagent from Invitrogen) at the time of injury. The microRNA mimic mediated up-regulation of miR-31 (**A**) and miR-21 (**B**) was confirmed by TaqMan assay-based real time PCR quantification on day 1 wound samples (* $p < 0.05$).

Supplementary Table S1: 57 differentially expressed high expresser microRNAs identified in paired skin and oral mucosal wound healing

				Skin								Oral								
				0hr		6hr		24hr		5day		0hr		6hr		24hr		5day		
Reporter Name	cluster	p-value	adj P-val	fold	SD	fold	SD	fold	SD	fold	SD	fold	SD	fold	SD	fold	SD	fold	SD	miR_seq
mmu-miR-31-5p	IV	6.15E-12	1.8492E-09	1	0.32025	0.66913	0.42253	0.84081	0.38847	11.6889	1.79791	22.8513	1.69253	18.3165	5.04849	21.6002	1.76514	33.9729	2.63708	AGGCAAGATGCTGGCATAGCT
mmu-miR-10a-5p	I	3.54E-11	7.0828E-09	1	0.13176	0.90841	0.35927	0.50246	0.15487	0.23832	0.0881	0.00482	0.00226	0.00693	0.00484	0.00965	0.00503	0.00747	0.00465	TACCCTGTAGATCCGAATTTGT
rno-miR-378a-3p	I	1.44E-09	1.7343E-07	1	0.0121	1.46934	0.51886	0.9391	0.13847	0.84576	0.08723	0.39645	0.02635	0.29053	0.01377	0.37856	0.02042	0.40841	0.03585	ACTGGACTTGGAGTCAGAAGGC
mmu-miR-223-3p	I	6.91E-09	4.6147E-07	1	0.18963	3.47255	1.04528	21.8819	7.09272	5.34173	2.79822	0.40893	0.03375	1.08788	0.35374	1.52225	1.00604	0.43993	0.07146	TGTCAGTTTGTCAAATACCCCAT
mmu-miR-146b-5p	I	1.30E-08	6.8495E-07	1	0.09845	1.35334	0.21642	1.7795	0.37526	1.27371	0.28653	0.16183	0.04531	0.2191	0.07541	0.36455	0.14836	0.28565	0.1048	TGAGAACTGAATTCCATAGGCTGT
mmu-miR-133a-3p	I	1.56E-07	3.6138E-06	1	0.56569	2.0078	1.05077	0.69721	0.31428	0.16211	0.01539	0.07094	0.03015	0.00977	0.0123	0.01411	0.01264	0.01421	0.00682	TTTGGTCCCCTTCAACCAGCTGT
mmu-miR-30a-5p	III	2.17E-07	4.1627E-06	1	0.10309	1.25145	0.11178	0.58288	0.0226	0.45316	0.03429	0.61118	0.02206	0.77524	0.21044	0.58071	0.0802	0.53593	0.03222	TGTAAACATCCTCGACTGGAAGCT
mmu-miR-1a-3p	I	2.67E-07	4.3449E-06	1	0.26741	2.96441	1.61244	0.751	0.07237	0.32537	0.08183	0.093	0.05463	0.02356	0.03125	0.02121	0.01363	0.04015	0.02132	TGGAATGTAAAGAAGTATGTAT
mmu-miR-195a-5p	III	5.67E-07	8.1159E-06	1	0.14822	1.08639	0.14093	0.61234	0.04451	0.43278	0.0546	0.9775	0.10956	0.88719	0.06351	0.86223	0.14369	0.64862	0.0468	TAGCAGCACAGAAATATTGGC
mmu-miR-206-3p	I	5.88E-07	8.2144E-06	1	0.39069	1.93417	0.49707	1.83228	0.36157	2.22764	0.81006	0.13447	0.10111	0.01284	0.01871	0.02617	0.01935	0.07181	0.03302	TGGAATGTAAGGAAGTGTGTGG
mmu-miR-23b-3p	V	1.16E-06	1.3914E-05	1	0.17943	0.71159	0.08563	0.63737	0.10603	0.49651	0.07132	1.17607	0.13128	0.86528	0.05485	1.21249	0.14399	1.1696	0.08045	ATCACATTGCCAGGGATTACC
mmu-miR-10b-5p	I	1.23E-06	1.4229E-05	1	0.15904	0.92243	0.40317	0.30263	0.09113	0.20605	0.06824	0.16388	0.0479	0.15016	0.04879	0.14704	0.03919	0.1517	0.02766	TACCCTGTAGAACCGAATTTGT
mmu-miR-21a-5p	II	1.67E-06	1.7127E-05	1	0.25392	1.99409	0.28009	4.56852	0.34421	8.70495	1.46603	0.96202	0.21269	1.93263	1.39535	3.38021	0.65147	3.00981	0.85541	TAGCTTATCAGACTGATGTTGA
mmu-miR-541-5p	I	1.82E-06	1.7935E-05	1	0.18335	1.35777	0.13839	1.09167	0.41589	5.42831	1.42195	0.69464	0.07466	0.71652	0.06905	0.87851	0.28299	1.61527	0.59453	AAGGGATTCTGATGTTGGTCACACT
mmu-miR-133b-3p	I	1.92E-06	1.8618E-05	1	1.05903	2.18718	1.39147	0.91693	0.4557	0.28245	0.03685	0.05764	0.03146	0.01127	0.01633	0.01133	0.00858	0.01373	0.00706	TTTGGTCCCCTTCAACCAGCT
mmu-miR-203-3p	V	2.34E-06	2.1713E-05	1	0.0321	0.7816	0.0757	0.62852	0.04755	0.56769	0.1051	1.19575	0.11079	1.47284	0.40683	1.13048	0.12823	1.29863	0.01301	TGAAATGTTTAGGACCACTAGT
mmu-miR-148b-3p	II	3.17E-06	2.7979E-05	1	0.12255	1.44014	0.17916	3.64374	0.78991	2.83251	0.21777	1.03422	0.10332	1.34953	0.41491	2.50508	1.00228	3.36653	0.65482	TCAGTGCATCACAGAACTTTGT
mmu-miR-199a-5p	IV	3.94E-06	3.1968E-05	1	0.05623	0.74372	0.14003	0.49441	0.11297	1.25568	0.26768	1.28942	0.13984	1.3851	0.15188	1.13168	0.10105	1.28514	0.15811	CCCAGTGTTCAGACTACCTGTTC
mmu-miR-200b-3p	V	4.74E-06	3.55E-05	1	0.13966	0.65697	0.14194	0.59729	0.03757	0.59844	0.13669	1.05491	0.08099	1.29823	0.19603	1.19178	0.06664	1.11726	0.05991	TAATACTGCCTGGTAATGATGAC
mmu-miR-125b-5p	V	9.08E-06	5.9968E-05	1	0.26157	0.60222	0.10091	0.31622	0.03516	0.49423	0.07592	1.13881	0.15729	0.94891	0.23488	0.64139	0.10051	0.73835	0.13935	TCCCTGAGACCCTAACTTGTGA
mmu-miR-30d-5p	III	1.62E-05	9.8884E-05	1	0.08011	1.08308	0.06138	0.90451	0.07191	0.67332	0.04992	0.86156	0.0467	0.87461	0.06623	0.74991	0.10542	0.69595	0.04463	TGTAAACATCCCCGACTGGAAGCT
mmu-miR-434-5p	I	1.63E-05	9.8884E-05	1	0.37043	1.85059	0.11366	0.62251	0.22646	1.96438	0.34935	0.56582	0.02515	0.81327	0.20492	0.58166	0.15055	0.67337	0.10784	GCTCGACTCATGGTTTGAACCA
rno-miR-25-3p	II	1.82E-05	0.00010713	1	0.16326	1.0748	0.09539	2.29016	0.47567	1.62575	0.06385	1.1716	0.06136	1.28665	0.19382	1.42755	0.20862	1.3511	0.01943	CATTGCACTTGTCTCGGTCTGA
mmu-miR-26a-5p	III	1.90E-05	0.00010944	1	0.2252	0.85487	0.11535	0.48225	0.03657	0.40368	0.05679	0.75499	0.09111	0.77771	0.09357	0.64725	0.12862	0.58426	0.03918	TTCAAGTAATCCAGGATAGGCT
mmu-miR-200c-3p	V	2.19E-05	0.00012071	1	0.21164	0.57869	0.19112	1.29397	0.20342	1.64047	0.17493	1.29407	0.20159	1.11953	0.27771	2.28466	0.75705	2.43074	0.24662	TAATACTGCCGGGTAATGATGGA
mmu-miR-126a-3p	IV	4.15E-05	0.00020936	1	0.02154	0.95076	0.14975	0.71711	0.08872	1.03941	0.05516	1.19991	0.07708	1.28318	0.14097	1.11501	0.11381	1.23767	0.10387	TCGTACCGTGAGTAATAATGC
mmu-let-7g-5p	II	4.45E-05	0.00021937	1	0.03802	1.32979	0.05651	1.86452	0.29297	1.17221	0.05641	1.08023	0.04972	1.30891	0.22111	1.44963	0.23806	1.57958	0.02899	TGAGGTAGTAGTTTGTACAGTT
mmu-miR-96-5p	III	5.14E-05	0.00024918	1	0.14515	1.05979	0.38404	0.3436	0.04575	0.22861	0.04905	0.5778	0.07657	0.72098	0.08036	0.69267	0.36034	0.3836	0.04451	TTTGGCACTAGCACATTTTTGCT
mmu-miR-99a-5p	III	9.23E-05	0.00039324	1	0.0842	1.5238	0.18286	0.87389	0.20862	0.53791	0.1482	1.14075	0.17856	1.38611	0.34808	1.0393	0.0855	1.01397	0.04929	AACCCGTAGATCCGATCTTGT
mmu-miR-27b-3p	V	1.06E-04	0.00043744	1	0.09228	1.07309	0.27389	0.65984	0.18401	0.74055	0.10806	1.24894	0.20612	1.78323	0.42364	1.4503	0.33778	1.56267	0.10899	TTCACAGTGGCTAAGTTCTGC
mmu-miR-34c-5p	IV	1.34E-04	0.00052527	1	0.35448	1.89039	0.31337	3.44168	0.59346	35.2178	29.9881	83.6016	69.0904	156.473	154.963	64.8819	77.0582	46.9643	36.9157	AGGCAGTGTAGTTAGCTGATTGC
mmu-miR-145a-5p	III	1.43E-04	0.00055157	1	0.32011	0.59084	0.13244	0.40661	0.03818	0.40204	0.08671	1.08829	0.337	0.50296	0.11037	0.96322	0.19515	0.75715	0.07126	GTCCAGTTTTCCCAGGAATCCCT
mmu-miR-200a-3p	III	1.80E-04	0.00065055	1	0.16103	1.1346	0.07371	1.07363	0.04588	0.48671	0.12516	0.77508	0.17721	1.43319	0.48086	0.95673	0.05542	0.75137	0.08551	TAACACTGTCTGGTAACGATGT
mmu-miR-181a-5p	V	2.43E-04	0.00082925	1	0.18853	0.78678	0.17649	0.7181	0.12254	0.53047	0.07622	1.18703	0.13501	0.97506	0.11242	0.78325	0.0767	0.83372	0.06517	AACATTCAACGCTGTCGGTGAGT
ptr-let-7i	II	2.63E-04	0.00088889	1	0.08981	1.07891	0.0828	1.52177	0.25506	1.62497	0.05519	0.84915	0.05696	0.88632	0.1746	1.09928	0.30967	1.42367	0.11814	TGAGGTAGTAGTTTGTGCTGTT
rno-miR-140-3p	II	3.03E-04	0.00099444	1	0.03248	0.81625	0.12978	2.93628	0.40461	1.61188	0.03768	2.39183	0.99652	2.03465	0.36358	2.40473	1.36428	2.47185	0.43058	ACCACAGGGTAGAACCACGGACT
mmu-miR-451a	IV	3.46E-04	0.00109958	1	0.30229	3.56606	0.83152	4.64544	1.287	6.47338	6.50628	20.1913	14.0438	12.3027	6.83623	20.7017	17.8385	9.41669	1.72506	AAACCGTTACCATTACTGAGT
mmu-miR-5099	II	3.88E-04	0.00120909	1	0.15874	1.27768	0.3366	3.36561	0.96627	1.60026	0.13534	2.97172	1.89256	3.99699	1.52294	4.23604	1.06287	1.92449	0.66428	TCTTAGATCGATGTGGTGCTC
mmu-miR-199b-3p	IV	3.99E-04	0.00123074	1	0.29356	1.33975	0.23079	1.21823	0.12986	1.74173	0.13536	1.61228	0.11655	2.08995	0.05308	1.78835	0.50672	2.02727	0.14601	ACAGTAGTCTGCACATTGGTT
mmu-miR-125a-5p	V	5.19E-04	0.00155201	1	0.33782	0.52121	0.10404	0.29036	0.03107	0.47078	0.09168	0.78488	0.10333	0.67794	0.23669	0.36058	0.09171	0.59685	0.14569	TCCCTGAGACCCTTTAACCTGT
mmu-miR-182-5p	V	5.89E-04	0.00170297	1	0.26868	0.70244	0.20781	0.44296	0.10253	0.35962	0.09244	0.81164	0.18349	0.93252	0.21908	0.82529	0.15139	0.86238	0.10079	TTTGGCAATGGTAGAACTCACACC
mmu-miR-127-3p	I	6.83E-04	0.00192812	1	0.45336	2.02466	0.51243	0.92469	0.27914	1.76644	0.37111	0.86383	0.20834	1.10595	0.11301	0.62443	0.08583	0.86403	0.08744	TCGGATCCGTCTGAGCTTGGCT
mmu-miR-145a-3p	V	7.82E-04	0.00216644	1	0.06649	1.32569	0.15052	0.67565	0.09031	0.64517	0.13044	1.06261	0.12131	1.22557	0.44141	0.91041	0.12037	0.99487	0.05221	GGATTCCTGGAAATACTGTTCT
mmu-miR-15b-5p	II	1.18E-03	0.00314941	1	0.25424	0.99616	0.15591	1.98649	0.28664	1.64861	0.23561	1.7898	0.52758	1.52272	0.1786	2.14746	0.45549	1.72315	0.34136	TAGCAGCACATCATGGTTTACA
mmu-miR-23a-3p	V	1.81E-03	0.00444036	1	0.16926	0.76725	0.12152	0.82237	0.10124	0.58281	0.08078	0.85269	0.20971	0.63712	0.08704	1.05739	0.20888	1.09479	0.10674	ATCACATTGCCAGGGATTTCC
mmu-let-7j	I	3.05E-03	0.00693415	1	0.34069	0.84994	0.1347	0.95187	0.19011	1.10822	0.06293	0.5864	0.12739	0.52593	0.1246	0.72003	0.17884	0.93691	0.08073	TGAGGTAGTAGTTTGTGCTGTTA
mmu-miR-24-3p	III	3.48E-03	0.00765341	1	0.22548	1.35166	0.24259	1.15112	0.16517	0.67155	0.13044	1.11421	0.20402	1.38102	0.21537	1.11682	0.19284	1.21367	0.11571	TGGCTCAGTTCAGCAGGAAC
mmu-miR-143-3p	III	4.15E-03	0.00873729	1	0.15358	1.3658	0.0735	0.92709	0.12281	0.79278	0.14447	1.23452	0.24076	1.58783	0.58506	1.08187	0.06926	1.34589	0.08416	TGAGATGAAGCACTGTAGCT
mmu-let-7f-5p	III	4.27E-03	0.00884356	1	0.04689	1.35267	0.142	1.13209	0.19693	0.90788	0.08043	0.92858	0.04582	1.13876	0.07414	0.97576	0.1223	1.07655	0.0986	TGAGGTAGTAGATTGTATAGTT
mmu-miR-126a-5p	IV	5.65E-03	0.01116063	1	0.14288	1.53879	0.27786	0.93601	0.32267	1.54215	0.28751	1.86448	0.28661	2.16946	0.51219	1.86865	0.72069	1.34598	0.29033	CATTATTACTTTTGGTACGCG
mmu-miR-205-5p	V	5.69E-03	0.01121347	1	0.14923	0.54933	0.10314	0.80858	0.16402	0.68718	0.06408	1.257	0.2574	0.93316	0.42272	1.18513	0.07672	1.10814	0.03802	TCCTTCATTCCACCGGAGTCTGT
mmu-miR-24-2-5p	IV	6.53E-03	0.01258097	1	0.04154	0.73266	0.05773	0.51965	0.12041	0.73674	0.07881	0.85222	0.09979	0.80685	0.06448	0.91514	0.33005	0.8398	0.01507	GTGCCTACTGAGCTGAAACAGT
mmu-miR-183-5p	V	6.83E-03	0.01302398	1	0.18994	0.83208	0.32582	0.75854	0.22133	0.5495	0.12943	0.87226	0.11367	0.871	0.16411	1.24506	0.1513	1.3214	0.18673	TATGGCACTGGTAGAATTCACT
mmu-let-7b-5p	V	7.44E-03	0.01397998	1	0.1229	0.7572	0.09032	1.02143	0.25084	0.83869	0.15729	1.18176	0.10922	1.00059	0.13623	1.25999	0.40817	1.57062	0.10129	TGAGGTAGTAGGTTGTGTGGTT
mmu-miR-152-3p	II	8.35E-03	0.0154895	1	0.21076	1.33151	0.11104	1.2382	0.1166	1.79904	0.04603	1.10854	0.02967	1.35951	0.30229	1.54318	0.59946	1.92556	0.15234	TCAGTGCATGACAGAACTTGG
mmu-miR-142a-5p	I	8.51E-03	0.01568941	1	0.02555	1.28481	0.26519	4.14374	0.44058	2.36219	0.34535	1.17246	0.26569	1.45481	1.13335	1.8446	1.5102	1.05501	0.29788	CCCATAAAGTAGAAAGCACTAC
mmu-miR-186-5p	III	9.29E-03	0.01681636	1	0.06908	1.14574	0.15497	0.58787	0.03325	0.66427	0.10696	0.84716	0.20868	0.82926	0.13423	0.9601	0.38089	0.65287	0.04752	CAAAGAATTCTCCTTTTGGGCT

miRNAs shown significant difference (p < 0.01), and have the number of reads > the average (5458) of the dataset.
adj P-val: P values adjusted with Benjamini-Hochberg procedure.
clusters were defined based on Hierarchical Clustering analysis using Morpheus.

Supplementary Table S2: Tissue-specific differentially expressed microRNAs in mouse skin and oral mucosal epithelium

miR	fold (oral/skin)	corr. q-val
mmu-miR-10b-5p	-1.982199442	3.55E-11
mmu-miR-451a	1.845365775	3.54E-10
mmu-miR-7211-3p	-1.743175942	5.49E-09
mmu-miR-10a-5p	-1.625557394	2.36E-08
mmu-miR-6540-5p	1.410432231	2.50E-08
mmu-miR-195a-3p	1.397298801	8.36E-08
mmu-miR-3103-5p	-1.374535577	2.82E-07
mmu-miR-665-5p	1.346613823	3.47E-07
mmu-miR-7082-5p	-1.196566429	5.24E-07
mmu-miR-6359	1.281108583	5.73E-07
mmu-miR-214-3p	1.269240053	1.01E-06
mmu-miR-7053-5p	1.352218936	1.13E-06
mmu-miR-6934-3p	1.325204188	1.35E-06
mmu-miR-678	-1.413271872	1.42E-06
mmu-miR-7647-3p	-1.342698653	1.63E-06
mmu-miR-7088-5p	-1.485014367	3.10E-06
mmu-miR-16-1-3p	1.337582778	3.60E-06
mmu-miR-7045-5p	-1.163735769	5.56E-06
mmu-miR-7082-3p	1.29298308	6.54E-06
mmu-miR-5104	-1.31362353	8.28E-06
mmu-miR-3473d	1.222056189	9.41E-06
mmu-miR-34b-3p	1.215255043	9.74E-06
mmu-miR-29c-5p	-1.483350303	1.24E-05
mmu-miR-150-5p	1.332006747	1.75E-05
mmu-miR-7022-3p	1.338666348	2.35E-05
mmu-miR-7075-5p	-1.446507597	2.37E-05
mmu-miR-676-3p	-1.199742321	3.65E-05
mmu-miR-7052-5p	-1.246722765	3.76E-05
mmu-miR-346-5p	1.430519097	4.41E-05
mmu-miR-146b-5p	-1.281482583	5.20E-05
mmu-miR-196a-5p	-1.284334608	5.54E-05
mmu-miR-101a-3p	-1.218546765	8.62E-05
mmu-miR-3070b-3p	-1.266188851	8.69E-05
mmu-miR-6942-5p	-1.348172231	0.000124
mmu-miR-1907	-1.421860348	0.000134
mmu-miR-705	-1.139990737	0.000137
mmu-miR-15a-3p	1.33767164	0.000196
mmu-miR-126a-3p	1.241031615	0.000212
mmu-miR-5112	-1.221503256	0.000223
mmu-miR-6931-5p	-1.177801676	0.000303
mmu-miR-6360	1.255440277	0.0004
mmu-miR-3473e	1.172623409	0.000427
mmu-miR-1983	1.176768986	0.000476
mmu-miR-467a-5p	1.165063814	0.000629
mmu-miR-374c-5p	-1.211108401	0.000634

mmu-miR-7235-5p	-1.3195672	0.000667
mmu-miR-375-3p	1.210377267	0.000668
mmu-miR-199a-3p	1.173644383	0.000686
mmu-miR-31-5p	1.146336437	0.000772
mmu-miR-365-3p	-1.230574533	0.000802
mmu-miR-138-2-3p	1.150686421	0.000809
mmu-let-7f-5p	-1.104261244	0.000827
mmu-miR-207	1.172299327	0.000877
mmu-miR-6980-5p	-1.177513002	0.001088
mmu-miR-1903	1.142199127	0.00113
mmu-miR-7665-5p	1.196517681	0.001178
mmu-miR-7028-5p	-1.205054435	0.00124
mmu-let-7k	-1.220767225	0.001302
mmu-miR-200c-3p	-1.090876142	0.001477
mmu-miR-6952-3p	1.141600262	0.001813
mmu-miR-3098-3p	1.157332636	0.001904
mmu-miR-7048-5p	-1.165290162	0.002081
mmu-miR-6996-5p	1.266197036	0.00211
mmu-miR-6958-3p	1.214933705	0.002136
mmu-miR-690	-1.076752067	0.002158
mmu-miR-7033-5p	-1.147956187	0.002453
mmu-miR-3569-3p	1.15329023	0.00252
mmu-miR-7578	1.267530421	0.002837
mmu-miR-3107-5p	1.158352835	0.002958
mmu-miR-466p-5p	-1.195193276	0.003021
mmu-miR-1187	1.083117192	0.003603
mmu-miR-6929-3p	1.138323233	0.003694
mmu-miR-6368	-1.142107201	0.003779
mmu-miR-181d-5p	1.20708225	0.003878
mmu-miR-7080-5p	-1.196021925	0.00391
mmu-miR-15b-5p	-1.12129573	0.004122
mmu-miR-6939-5p	-1.175468921	0.004307
mmu-miR-98-5p	-1.165509765	0.004612
mmu-miR-7049-3p	1.237488124	0.005555
mmu-miR-214-5p	1.23282749	0.006668
mmu-miR-7034-5p	1.140768613	0.006939
mmu-let-7a-5p	-1.066357154	0.007239
mmu-miR-7211-5p	1.155266855	0.008024
mmu-miR-7225-3p	1.122008402	0.008558
mmu-miR-744-5p	-1.211651849	0.009022

MicroRNA expression was measured using a mouse MicroRNA Microarray (LC Sciences) on pooled RNA samples isolated from mouse skin and oral mucosa samples (n=3).
Differential expression analysis was performed with CyberT using a VSN normalization procedure.
Multiple hypothesis testing correction is performed with Bonferroni procedure, and Bonferroni corrected q-values were presended.
Cut-off q-value of 0.01 was used.

Supplementary Table S3: Validation of the tissue specificity of 57 differentially expressed high expresser microRNAs with independent miR profiling experiments

			Original Dataset **	Validation Dataset***
Index	Reporter Name	cluster *	tissue specific	tissue specific
80	mmu-miR-10a-5p	I	1	1
1	rno-miR-378a-3p	I	1	x
38	mmu-miR-223-3p	I	1	no match
65	mmu-miR-146b-5p	I	1	1
73	mmu-miR-133a-3p	I	1	x
48	mmu-miR-1a-3p	I	1	x
42	mmu-miR-206-3p	I	x	x
79	mmu-miR-10b-5p	I	1	1
11	mmu-miR-541-5p	I	x	no match
72	mmu-miR-133b-3p	I	x	x
17	mmu-miR-434-5p	I	x	no match
74	mmu-miR-127-3p	I	x	no match
82	mmu-let-7j	I	x	x
70	mmu-miR-142a-5p	I	x	no match
41	mmu-miR-21a-5p	II	x	x
63	mmu-miR-148b-3p	II	x	x
2	rno-miR-25-3p	II	x	x
83	mmu-let-7g-5p	II	x	x
5	ptr-let-7i	II	x	x
3	rno-miR-140-3p	II	x	x
12	mmu-miR-5099	II	x	x
58	mmu-miR-15b-5p	II	x	1
59	mmu-miR-152-3p	II	x	x
28	mmu-miR-30a-5p	III	1	x
51	mmu-miR-195a-5p	III	x	x
25	mmu-miR-30d-5p	III	x	x
33	mmu-miR-26a-5p	III	x	x
8	mmu-miR-96-5p	III	1	no match
7	mmu-miR-99a-5p	III	x	x
67	mmu-miR-145a-5p	III	x	x
47	mmu-miR-200a-3p	III	x	x
34	mmu-miR-24-3p	III	x	x
69	mmu-miR-143-3p	III	x	x
84	mmu-let-7f-5p	III	x	1
53	mmu-miR-186-5p	III	x	x
23	mmu-miR-31-5p	IV	1	1
50	mmu-miR-199a-5p	IV	x	x
76	mmu-miR-126a-3p	IV	x	1
21	mmu-miR-34c-5p	IV	1	no match
16	mmu-miR-451a	IV	1	1
49	mmu-miR-199b-3p	IV	x	1
75	mmu-miR-126a-5p	IV	1	no match
35	mmu-miR-24-2-5p	IV	x	x
36	mmu-miR-23b-3p	V	x	x
44	mmu-miR-203-3p	V	x	x
46	mmu-miR-200b-3p	V	x	x
77	mmu-miR-125b-5p	V	x	x
45	mmu-miR-200c-3p	V	x	1
30	mmu-miR-27b-3p	V	x	x
56	mmu-miR-181a-5p	V	x	x
78	mmu-miR-125a-5p	V	x	x
55	mmu-miR-182-5p	V	x	x
68	mmu-miR-145a-3p	V	x	no match
37	mmu-miR-23a-3p	V	x	x
43	mmu-miR-205-5p	V	x	x
54	mmu-miR-183-5p	V	x	x
88	mmu-let-7b-5p	V	x	x

miRNAs shown significant difference ($p < 0.01$), and have the number of reads > the average (5458) of the dataset.

*: clusters were defined based on Hierarchical Clustering analysis using Morpheus.

**: Original dataset:Tissue-specific differential expressed miRs were defined in [Sci. Rep. (2019) 9:7160].

***: Validation dataset 1: Tissue-specific differential expressed miRs were identifed by microarray analysis using paired skin and oral mucosal tissue samples from mice.

Supplementary Table S4: Statistical analysis on differential expression of selected microRNAs

Cluster I

mmu-miR-10b-5p

miR-Seq

hour	skin	oral	stdev (s)	stdev (o)
0	1	0.16388	0.15904	0.0479
6	0.92243	0.15016	0.40317	0.04879
24	0.30263	0.14704	0.09113	0.03919
120	0.20605	0.1517	0.06824	0.02766

one-way ANOVA (skin) p-value = 0.0042662840
one-way ANOVA (oral) p-value = 0.9613844771

two-way ANOVA p-value = 3.514122e-6

LCM/TaqMan

hour	skin	oral	stdev (s)	stdev (o)
0	1.05813	0.05347	0.4068	0.01685
6	1.33081	0.03662	0.48886	0.02027
24	0.42458	0.08254	0.31821	0.08102
120	0.27895	0.04671	0.18151	0.0611

one-way ANOVA (skin) p-value = 0.0000004779
one-way ANOVA (oral) p-value = 0.4325128555

two-way ANOVA p-value = 1.951684e-8

mmu-miR-223-3p

miR-Seq

hour	skin	oral	stdev (s)	stdev (o)
0	0.999953	0.408908	0.189619	0.033747
6	3.472382	1.087826	1.045229	0.353727
24	21.88087	1.522174	7.092387	1.005989
120	5.341474	0.439913	2.798091	0.07146

one-way ANOVA (skin) p-value = 0.0006251456
one-way ANOVA (oral) p-value = 0.0924204518

two-way ANOVA p-value = 1.071586e-5

LCM/TaqMan

hour	skin	oral	stdev (s)	stdev (o)
0	1.00479	0.22033	0.1055	0.1498
6	6.96063	4.32524	3.43335	2.17314
24	90.333	0.82264	22.3316	0.14561
120	71.283	0.46588	36.6742	0.11096

one-way ANOVA (skin) p-value = 0.0005539250
one-way ANOVA (oral) p-value = 0.0000051701

two-way ANOVA p-value = 1.393415e-6

Cluster II

mmu-miR-21-5p

miR-Seq

hour	skin	oral	stdev (s)	stdev (o)
0	1.000012	0.962027	0.253921	0.212693
6	1.99411	1.932654	0.280097	1.395369
24	4.56857	3.380248	0.344211	0.651479
120	8.705052	3.00985	1.466047	0.855424

one-way ANOVA (skin) p-value = 0.0000085387
one-way ANOVA (oral) p-value = 0.0381469755

two-way ANOVA p-value = 1.008347e-4

LCM/TaqMan

hour	skin	oral	stdev (s)	stdev (o)
0	1.1171	0.31085	0.54907	0.2744
6	0.94479	0.13192	0.34645	0.07162
24	1.61073	1.26236	0.47516	1.3955
120	4.80132	1.32614	2.10515	0.79613

one-way ANOVA (skin) p-value = 0.0000000000
one-way ANOVA (oral) p-value = 0.0070474133

two-way ANOVA p-value = 0.000000e+0

mmu-miR-140-3p

miR-Seq

hour	skin	oral	stdev (s)	stdev (o)
0	1.000043	2.391934	0.032481	0.996564
6	0.816283	2.034733	0.129787	0.363597
24	2.936411	2.40483	0.404628	1.364335
120	1.611947	2.471958	0.037683	0.4306

one-way ANOVA (skin) p-value = 0.0000077748
one-way ANOVA (oral) p-value = 0.9286297066

two-way ANOVA p-value = 0.01341338

LCM/TaqMan

hour	skin	oral	stdev (s)	stdev (o)
0	1	0.37114	0.93355	0.3016
6	3.7718	1.27243	2.34304	0.46806
24	6.80201	0.48988	5.23345	0.22643
120	5.65733	1.41787	4.49115	0.31332

one-way ANOVA (skin) p-value = 0.0024882129
one-way ANOVA (oral) p-value = 0.0000007733

two-way ANOVA p-value = 8.485368e-11

Cluster III

mmu-miR-99a-5p

miR-Seq

hour	skin	oral	stdev (s)	stdev (o)
0	1	1.140749	0.0842	0.178556
6	1.5238	1.386114	0.182855	0.348079
24	0.873889	1.039303	0.208621	0.085498
120	0.537912	1.013975	0.148197	0.049289

one-way ANOVA (skin) p-value = 0.0005390556
one-way ANOVA (oral) p-value = 0.1752214836

LCM/TaqMan

hour	skin	oral	stdev (s)	stdev (o)
0	1.03602	2.7167	0.28881	1.63517
6	3.26437	2.85863	1.77585	1.516
24	0.9841	1.10762	0.39102	0.34286
120	1.14221	1.47977	0.1581	0.40894

one-way ANOVA (skin) p-value = 0.0168764875
one-way ANOVA (oral) p-value = 0.1988154933

two-way ANOVA p-value = 4.689403e-2

two-way ANOVA p-value = 1.579563e-1

mmu-miR-26a-5p

miR-Seq

hour	skin	oral	stdev (s)	stdev (o)
0	1	0.75499	0.225197	0.091114
6	0.854867	0.777713	0.115354	0.09357
24	0.482248	0.647254	0.036568	0.12862
120	0.403676	0.58426	0.056791	0.039178

one-way ANOVA (skin) p-value = 0.0013458458
one-way ANOVA (oral) p-value = 0.1059181021

two-way ANOVA p-value = 9.013084e-1

LCM/TaqMan

hour	skin	oral	stdev (s)	stdev (o)
0	1.00351	0.70583	0.09241	0.11635
6	1.40049	0.49348	0.21375	0.06119
24	0.21055	0.1531	0.1158	0.01774
120	0.47231	0.63376	0.14798	0.05709

one-way ANOVA (skin) p-value = 0.0000000000
one-way ANOVA (oral) p-value = 0.0000000001

two-way ANOVA p-value = 5.532423e-10

Cluster IV

mmu-miR-31-5p

miR-Seq

hour	skin	oral	stdev (s)	stdev (o)
0	0.999445	22.83856	0.32007	1.691587
6	0.668761	18.30634	0.422295	5.04569
24	0.840345	21.58825	0.388254	1.764157
120	11.68245	33.95408	1.796909	2.635619

one-way ANOVA (skin) p-value = 0.0000012741
one-way ANOVA (oral) p-value = 0.0013593949

two-way ANOVA p-value = 2.132738e-13

LCM/TaqMan

hour	skin	oral	stdev (s)	stdev (o)
0	1.03405	6.83589	0.27175	2.32593
6	0.45834	10.0726	0.21271	1.36538
24	0.20257	8.63327	0.05702	0.95355
120	17.5665	46.2521	4.16547	24.8059

one-way ANOVA (skin) p-value = 0.0000000001
one-way ANOVA (oral) p-value = 0.0000422971

two-way ANOVA p-value = 1.503829e-5

mmu-miR-451a

miR-Seq

hour	skin	oral	stdev (s)	stdev (o)
0	1.000601	20.20339	0.302476	14.05226
6	3.568201	12.31014	0.832018	6.840336
24	4.648233	20.71412	1.287771	17.84923
120	6.477267	9.422348	6.510191	1.726098

one-way ANOVA (skin) p-value = 0.3112343244
one-way ANOVA (oral) p-value = 0.5880771974

two-way ANOVA p-value = 4.604120e-3

LCM/TaqMan

hour	skin	oral	stdev (s)	stdev (o)
0	1	8.52394	0.52108	7.36411
6	75.7683	39.9092	59.1761	3.87191
24	42.3235	101.572	53.6771	66.6428
120	67.436	240.362	73.0032	141.223

one-way ANOVA (skin) p-value = 0.0495521565
one-way ANOVA (oral) p-value = 0.0000203422

two-way ANOVA p-value = 1.102023e-1

mmu-miR-34c-5p

miR-Seq

hour	skin	oral	stdev (s)	stdev (o)
0	1.0029	83.8442	0.35551	69.2909
6	1.89588	156.927	0.31428	155.413
24	3.45167	65.0702	0.59518	77.2819
120	35.32	47.1006	30.0751	37.0228

one-way ANOVA (skin) p-value = 0.0628504365
one-way ANOVA (oral) p-value = 0.5435316838

two-way ANOVA p-value = 1.296908e-2

LCM/TaqMan

hour	skin	oral	stdev (s)	stdev (o)
0	1.17721	64.2194	0.74403	56.8412
6	1.5443	82.8872	0.4456	55.0774
24	2.96769	35.3505	1.53904	41.0694
120	14.4752	162.254	2.07662	48.2243

one-way ANOVA (skin) p-value = 0.0000000000
one-way ANOVA (oral) p-value = 0.0022578420

two-way ANOVA p-value = 1.307810e-9

mmu-miR-24-2-5p

miR-Seq

hour	skin	oral	stdev (s)	stdev (o)
0	1.00001	0.85222	0.04154	0.09979
6	0.73267	0.80686	0.05773	0.06448
24	0.51965	0.91515	0.12041	0.33005
120	0.73675	0.83981	0.07881	0.01507

one-way ANOVA (skin) p-value = 0.0006448633
one-way ANOVA (oral) p-value = 0.8934766793

two-way ANOVA p-value = 7.469976e-2

LCM/TaqMan

hour	skin	oral	stdev (s)	stdev (o)
0	1.02554	0.68663	0.24149	0.18912
6	0.82815	0.64816	0.05124	0.09644
24	0.71884	0.4451	0.20117	0.0765
120	0.53665	0.80411	0.0627	0.3268

one-way ANOVA (skin) p-value = 0.0004074361
one-way ANOVA (oral) p-value = 0.0378170837

two-way ANOVA p-value = 1.629529e-2

mmu-miR-126a-5p

miR-Seq

	skin	oral	stdev (s)	stdev (o)

LCM/TaqMan

	skin	oral	stdev (s)	stdev (o)

hour	0	0.9999	1.86429	0.14287	0.28658
	6	1.53863	2.16924	0.27783	0.51213
	24	0.93591	1.86845	0.32264	0.72061
	120	1.54199	1.34584	0.28748	0.2903

one-way ANOVA (skin) p-value = 0.0367424477
one-way ANOVA (oral) p-value = 0.2915927220

two-way ANOVA p-value = 3.080962e-3

hour	0	1.02281	1.13154	0.23304	0.61011
	6	0.82478	0.74533	0.21692	0.09552
	24	0.60181	0.50036	0.18889	0.26269
	120	0.75244	0.90493	0.17029	0.13916

one-way ANOVA (skin) p-value = 0.0153397840
one-way ANOVA (oral) p-value = 0.0311976849

two-way ANOVA p-value = 8.064029e-1

mmu-miR-126a-3p

miR-Seq

		skin	oral	stdev (s)	stdev (o)
hour	0	1.00002	1.19993	0.02154	0.07708
	6	0.95078	1.28321	0.14976	0.14097
	24	0.71712	1.11503	0.08872	0.11381
	120	1.03943	1.23769	0.05516	0.10387

one-way ANOVA (skin) p-value = 0.0107302984
one-way ANOVA (oral) p-value = 0.3604404602

two-way ANOVA p-value = 4.500348e-6

LCM/TaqMan

		skin	oral	stdev (s)	stdev (o)
hour	0	1.0121	1.08732	0.17104	0.33538
	6	1.07103	1.06201	0.26453	0.1185
	24	0.75819	0.59221	0.07656	0.19267
	120	0.84919	0.99827	0.04835	0.3132

one-way ANOVA (skin) p-value = 0.0126603858
one-way ANOVA (oral) p-value = 0.0101819129

two-way ANOVA p-value = 8.434429e-1

mmu-miR-199a-5p

miR-Seq

		skin	oral	stdev (s)	stdev (o)
hour	0	1	1.28941	0.05623	0.13984
	6	0.74372	1.38509	0.14003	0.15188
	24	0.49441	1.13167	0.11297	0.10105
	120	1.25567	1.28514	0.26768	0.15811

one-way ANOVA (skin) p-value = 0.0024600154
one-way ANOVA (oral) p-value = 0.2449372673

two-way ANOVA p-value = 8.279114e-6

LCM/TaqMan

		skin	oral	stdev (s)	stdev (o)
hour	0	1.03981	1.66483	0.31694	0.80451
	6	1.0016	1.03396	0.1058	0.22706
	24	0.78837	0.58865	0.12739	0.10116
	120	1.40373	1.11833	0.51087	0.25234

one-way ANOVA (skin) p-value = 0.0215631834
one-way ANOVA (oral) p-value = 0.0041670237

two-way ANOVA p-value = 6.975289e-1

mmu-miR-199b-3p

miR-Seq

		skin	oral	stdev (s)	stdev (o)
hour	0	1.00002	1.61231	0.29356	0.11655
	6	1.33978	2.08999	0.2308	0.05308
	24	1.21826	1.78838	0.12986	0.50673
	120	1.74177	2.02731	0.13536	0.14602

one-way ANOVA (skin) p-value = 0.0144157380
one-way ANOVA (oral) p-value = 0.1954840301

two-way ANOVA p-value = 3.915706e-5

LCM/TaqMan

		skin	oral	stdev (s)	stdev (o)
hour	0	1.00866	1.53192	0.14737	0.42251
	6	0.93754	0.90036	0.20295	0.09239
	24	1.03203	0.60616	0.17864	0.18131
	120	0.92642	0.87223	0.11186	0.05888

one-way ANOVA (skin) p-value = 0.6181493984
one-way ANOVA (oral) p-value = 0.0000120929

two-way ANOVA p-value = 9.796490e-1

Cluster V

mmu-miR-125b-5p

miR-Seq

		skin	oral	stdev (s)	stdev (o)
hour	0	1.000001	1.138808	0.261571	0.157288
	6	0.602217	0.948907	0.100906	0.23488
	24	0.316224	0.641391	0.035158	0.100507
	120	0.494231	0.738349	0.075924	0.139347

one-way ANOVA (skin) p-value = 0.0026510959
one-way ANOVA (oral) p-value = 0.0250541501

two-way ANOVA p-value = 7.743095e-4

LCM/TaqMan

		skin	oral	stdev (s)	stdev (o)
hour	0	1.0807	1.81417	0.38263	0.6528
	6	1.09994	0.33573	0.31694	0.13976
	24	0.27759	0.74107	0.27759	0.37983
	120	1.04861	0.86166	0.40312	0.42776

one-way ANOVA (skin) p-value = 0.0000006328
one-way ANOVA (oral) p-value = 0.0000533232

two-way ANOVA p-value = 3.550410e-1

mmu-miR-125a-5p

miR-Seq

		skin	oral	stdev (s)	stdev (o)
hour	0	0.999995	0.784881	0.337814	0.103334
	6	0.521206	0.677937	0.104037	0.236693
	24	0.290362	0.360574	0.031068	0.091706
	120	0.470782	0.596849	0.091684	0.145685

one-way ANOVA (skin) p-value = 0.0080004410

LCM/TaqMan

		skin	oral	stdev (s)	stdev (o)
hour	0	1.13827	0.73804	0.50428	0.18046
	6	1.96972	1.24938	1.08421	0.37058
	24	0.19846	0.13364	0.13421	0.06417
	120	1.39059	0.99888	0.90191	0.10161

one-way ANOVA (skin) p-value = 0.0034683330

one-way ANOVA (oral) p-value = 0.0505159562

two-way ANOVA p-value = 6.257289e-1

one-way ANOVA (oral) p-value = 0.0000341469

two-way ANOVA p-value = 1.078114e-3

Supplementary Table S5: Statistical analysis on wound closure

A: in vitro wounds treated with control and miR-21 mimic

A.1: 2way ANOVA - in vitro wounds treated with control and miR-21 mimic

Two-way ANOVA	Ordinary					
Alpha	0.05					
ANOVA table	SS	DF	MS	F (DFn, DFd)	P value	Significant?
Interaction	688	3	229.3	F (3, 18) = 3.006	P = 0.0575	No
Row Factor (time)	12361	3	4120	F (3, 18) = 54.01	P < 0.0001	Yes
Column Factor (treatment)	6790	1	6790	F (1, 18) = 89.00	P < 0.0001	Yes
Residual	1373	18	76.29			

A.2: Bonferroni's multiple comparisons test - in vitro wounds treated with control and miR-21 mimic

		Mean (ctrl)	Mean (miR-21)	Mean Diff.	95% CI of diff.	Significant?	Summary	Adjusted P Value
day 1	Row 1	15.49	32.27	-16.78	-35.29 to 1.727	No	ns	0.0864
day 2	Row 2	17.44	64.13	-46.69	-68.81 to -24.57	Yes	****	< 0.0001
day 3	Row 3	53.88	90.14	-36.26	-54.77 to -17.75	Yes	***	0.0001
day 4	Row 4	58.83	91.54	-32.71	-51.21 to -14.20	Yes	***	0.0005

B: in vitro wounds treated with control and miR-31 mimic

B.1: 2way ANOVA - in vitro wounds treated with control and miR-31 mimic

Two-way ANOVA	Ordinary					
Alpha	0.05					
ANOVA table	SS	DF	MS	F (DFn, DFd)	P value	Significant?
Interaction	1579	3	526.3	F (3, 22) = 10.79	P = 0.0001	Yes
Row Factor (time)	12314	3	4105	F (3, 22) = 84.16	P < 0.0001	Yes
Column Factor (treatment)	16346	1	16346	F (1, 22) = 335.2	P < 0.0001	Yes
Residual	1073	22	48.77			

B.2: Bonferroni's multiple comparisons test - in vitro wounds treated with control and miR-31 mimic

		Mean (ctrl)	Mean (miR-31)	Mean Diff.	95% CI of diff.	Significant?	Summary	Adjusted P Value
day 1	Row 1	15.49	45.93	-30.44	-43.87 to -17.01	Yes	****	< 0.0001
day 2	Row 2	17.44	91.49	-74.05	-90.50 to -57.60	Yes	****	< 0.0001
day 3	Row 3	53.88	100	-46.12	-59.55 to -32.69	Yes	****	< 0.0001
day 4	Row 4	58.83	100	-41.17	-54.60 to -27.73	Yes	****	< 0.0001

C: in vivo murine skin wounds treated with control and miR-21 mimic

C.1: 2way ANOVA - in vivo murine skin wounds treated with control and miR-21 mimic

Two-way ANOVA	Ordinary					
Alpha	0.05					
ANOVA table	SS	DF	MS	F (DFn, DFd)	P value	Significant?
Interaction	137.4	9	15.27	F (9, 90) = 0.3972	P = 0.9335	No
Row Factor (time)	78932	9	8770	F (9, 90) = 228.1	P < 0.0001	Yes
Column Factor (treatment)	259.5	1	259.5	F (1, 90) = 6.751	P = 0.0109	Yes
Residual	3460	90	38.44			

C.2: Bonferroni's multiple comparisons test - in vivo murine skin wounds treated with control and miR-21 mimic

		Mean (ctrl)	Mean (miR-21)	Mean Diff.	95% CI of diff.	Significant?	Summary	Adjusted P Value
day 1	Row 1	15.91	18.62	-2.708	-13.51 to 8.097	No	ns	> 0.9999
day 2	Row 2	27.35	33.92	-6.57	-17.37 to 4.235	No	ns	0.8354
day 3	Row 3	40.3	40.68	-0.3766	-11.18 to 10.43	No	ns	> 0.9999
day 4	Row 4	51.2	52.35	-1.146	-11.95 to 9.659	No	ns	> 0.9999
day 5	Row 5	58.17	61.45	-3.28	-14.08 to 7.525	No	ns	> 0.9999
day 6	Row 6	61.85	68.09	-6.237	-17.04 to 4.567	No	ns	> 0.9999
day 7	Row 7	70.57	76.67	-6.107	-16.91 to 4.698	No	ns	> 0.9999
day 8	Row 8	92.89	94.15	-1.26	-12.06 to 9.545	No	ns	> 0.9999
day 9	Row 9	96.26	97.7	-1.432	-12.24 to 9.373	No	ns	> 0.9999
day 10	Row 10	97.51	99.25	-1.732	-12.54 to 9.073	No	ns	> 0.9999

D: in vivo murine skin wounds treated with control and miR-31 mimic

D.1: 2way ANOVA - in vivo murine skin wounds treated with control and miR-31 mimic

Two-way ANOVA	Ordinary					
Alpha	0.05					
ANOVA table	SS	DF	MS	F (DFn, DFd)	P value	Significant?
Interaction	2000	9	222.2	F (9, 100) = 5.096	P < 0.0001	Yes
Row Factor (time)	66462	9	7385	F (9, 100) = 169.4	P < 0.0001	Yes
Column Factor (treatment)	4887	1	4887	F (1, 100) = 112.1	P < 0.0001	Yes
Residual	4360	100	43.6			

D.2: Bonferroni's multiple comparisons test - in vivo murine skin wounds treated with control and miR-31 mimic

		Mean (ctrl)	Mean (miR-31)	Mean Diff.	95% CI of diff.	Significant?	Summary	Adjusted P Value
day 1	Row 1	15.91	34.05	-18.14	-29.08 to -7.194	Yes	****	< 0.0001
day 2	Row 2	27.35	54.9	-27.54	-38.48 to -16.60	Yes	****	< 0.0001
day 3	Row 3	40.3	63.57	-23.26	-34.21 to -12.32	Yes	****	< 0.0001
day 4	Row 4	51.2	65.9	-14.7	-25.64 to -3.753	Yes	**	0.002
day 5	Row 5	58.17	68.4	-10.23	-21.18 to 0.7104	No	ns	0.0851
day 6	Row 6	61.85	75.37	-13.52	-24.46 to -2.574	Yes	**	0.006
day 7	Row 7	70.57	82.33	-11.77	-22.71 to -0.8251	Yes	*	0.0262
day 8	Row 8	92.89	96.39	-3.495	-14.44 to 7.449	No	ns	> 0.9999
day 9	Row 9	96.26	99.05	-2.787	-13.73 to 8.157	No	ns	> 0.9999
day 10	Row 10	97.51	99.7	-2.187	-13.13 to 8.757	No	ns	> 0.9999

Supplementary Table S6a: Bioinformatics prediction of pathways targeted by mmu-miR-31-3p

KEGG pathway	p-value	#genes targeted	gene targeted
Steroid biosynthesis	1.22E-08	1	Dhcr24
Steroid hormone biosynthesis	4.39E-07	1	Hsd3b1
Vitamin B6 metabolism	0.00946592	1	Sh3bp1
Thiamine metabolism	0.00948411	1	Tpk1
Adrenergic signaling in cardiomyocytes	0.00948411	3	Ppp2r2a, Cacnb2, Ppp2r3c
Sphingolipid metabolism	0.02507051	2	Sgpp2, B4galt6
B cell receptor signaling pathway	0.02624945	3	Vav3, Cd72, Ppp3ca
MAPK signaling pathway	0.03228995	6	Mef2c, Map3k1, Cacnb2, Dusp7, Rasa1, Ppp3ca
Taurine and hypotaurine metabolism	0.04608911	1	Gad2
Gap junction	0.04672907	1	Gjd2
Ovarian steroidogenesis	0.04672907	1	Hsd3b1
mRNA surveillance pathway	0.04845908	4	Ppp2r2a, Cpsf2, Ppp2r3c, Nxf1
Axon guidance	0.04845908	4	Rasa1, Ppp3ca, Epha7, Sema6c

MicroRNA targeted pathway analysis were performed using DIANA-mirPath (v3.0), with microT-CDS as microRNA target prediction method (microT threshold = 0.8
A cut off p value of 0.05 was used.

Supplementary Table S6b: Bioinformatics prediction of pathways targeted by Cluster IV microRNAs

KEGG pathway	p-value	#microRNAs	#genes targeted	microRNA	gene
Steroid hormone biosynthesis	7.68E-20	3	10		
				mmu-miR-126a-5p	Ugt1a6b, Ugt1a2, Ugt1a9, Ugt1a10, Ugt1a7c, Ugt1a5, Ugt1a1, Ugt1a6a
				mmu-miR-31-5p	Hsd3b1
				mmu-miR-34c-5p	Cyp2c54
Drug metabolism - cytochrome P450	5.25E-05	1	8		
				mmu-miR-126a-5p	Ugt1a6b, Ugt1a2, Ugt1a9, Ugt1a10, Ugt1a7c, Ugt1a5, Ugt1a1, Ugt1a6a
Dorso-ventral axis formation	0.00010201	5	10		
				mmu-miR-34c-5p	Notch1, Map2k1, Notch2, Notch4
				mmu-miR-24-2-5p	Kras
				mmu-miR-199b-3p	Cpeb3
				mmu-miR-126a-5p	Cpeb2, Sos1
				mmu-miR-199a-5p	Sos2, Ets1
Gap junction	0.00062218	6	9		
				mmu-miR-34c-5p	Map2k1, Grm1
				mmu-miR-126a-5p	Sos1, Map3k2
				mmu-miR-199b-3p	Plcb1, Gja1
				mmu-miR-31-5p	Gjd2
				mmu-miR-199a-5p	Sos2
				mmu-miR-24-2-5p	Kras
Proteoglycans in cancer	0.00062218	6	23		
				mmu-miR-34c-5p	Map2k1, Camk2b, Araf, Met, Rras
				mmu-miR-199a-5p	Tgfb2, Sos2, Wnt7b, Fzd10, Fzd4, Fzd5
				mmu-miR-126a-5p	Erbb4, Wnt16, Ank3, Sos1, Cd44
				mmu-miR-199b-3p	Cblb, Igf1, Erbb4, Arhgef12, Fn1, Met, Mtor
				mmu-miR-24-2-5p	Kras
				mmu-miR-31-5p	Fzd3
AMPK signaling pathway	0.00381211	7	18		
				mmu-miR-34c-5p	Irs4, Ppp2r3a, Creb1, Ppp2r5a
				mmu-miR-24-2-5p	Ppp2r5c
				mmu-miR-199a-5p	Rab10, Slc2a4, Stradb, Prkag2, Sirt1
				mmu-miR-199b-3p	Scd2, Ppp2r2a, Igf1, Scd4, Mtor
				mmu-miR-31-5p	Ppp2r2a, Ppp2r3c, Sirt1

				mmu-miR-126a-5p	Ppargc1a
				mmu-miR-126a-3p	Irs1
Wnt signaling pathway	0.00682663	5	16		
				mmu-miR-31-5p	Fzd3, Ppp3ca
				mmu-miR-126a-5p	Axin1, Wnt16, Cxxc4, Ppp3ca
				mmu-miR-199b-3p	Csnk1a1, Plcb1, Mapk[illegible]
				mmu-miR-199a-5p	Wnt7b, Gsk3b, Fzd10, Fzd4, Fzd5
				mmu-miR-34c-5p	Lef1, Camk2b, Daam1
Ascorbate and aldarate metabolism	0.00721958	1	8		
				mmu-miR-126a-5p	Ugt1a6b, Ugt1a2, Ugt1a9, Ugt1a10, Ugt1a7c, Ugt1a5, Ugt1a1, Ugt1a6[illegible]

MicroRNA targeted pathway analysis were performed using DIANA-mirPath (v3.0), with microT-CDS as microRNA target prediction method (microT threshold = 0.[illegible]
A cut off p value of 0.01 was used.
